# Ophthalmological outcomes of unilateral coronal synostosis in young children

**DOI:** 10.1186/s12886-020-01547-1

**Published:** 2020-08-04

**Authors:** Wen-Ting Luo, Xin Chen, Yi-Dan Zhang, Qing-Yu Liu, Tong Qiao

**Affiliations:** Shanghai Children’s Hospital, Shanghai Jiao Tong University, Shanghai, 200062 China

**Keywords:** Unilateral coronal synostosis, Superior oblique muscle palsy, Monocular elevator deficiency, Forced duction test, Standard Knapp procedure

## Abstract

**Background:**

To report refractive outcomes, describe types of strabismus and evaluate the outcomes of surgical intervention for unilateral coronal synostosis (UCS) in paediatric patients.

**Methods:**

This study retrospectively included 30 UCS cases. Patients aged from 3 months to 6 years (median: 1.8 years) were enrolled from January 2018 to December 2019 at Shanghai Children’s Hospital. Sixteen patients had all types of strabismus; 15 of these patients underwent surgery.

**Results:**

Refractive errors of 30 cases were included. In 60% of patients, astigmatism of 1.00D or more existed in not less than one eye at last record. Twenty (66.7%) patients had the larger amount of astigmatism in the contralateral eye. Fifteen patients received strabismus surgery, of whom 6 patients with monocular elevation deficiency (MED) underwent the standard Knapp procedure, with or without a horizontal deviation procedure. Fifteen cases were horizontally aligned within 5 prism dioptres (Δ). Six patients with MED (100%) had attained ≥25% elevation improvement after surgery, and the vertical deviation decreased from 25.83 Δ ± 4.92 Δ (range, 20 Δ-30 Δ) to 0.83 Δ ± 4.92 Δ after surgery (range, 0 Δ-10 Δ), for an improvement of 26.67 Δ ± 4.08 Δ (*t* = 16 *P* < 0.05). In 1 patient with esotropia, the horizontal deviation decreased from + 80 Δ to + 5 Δ after surgery. One patient was diagnosed with trichiasis and one with contralateral lacrimal duct obstruction.

**Conclusions:**

Contralateral MED was also the main type of strabismus in UCS. Superior oblique muscle palsy was still the most common, as previously reported. There is a risk of developing a higher astigmatism and anisometropia in the contralateral eye to synostosis. Other ophthalmic disorders should be treated in a timely manner.

**Trial registration:**

The study was approved by the Institutional Review Board of Shanghai Children’s Hospital (approval No. 2020R023-E01) and adhered to the tenets of the Declaration of Helsinki. Ethics approval was procured on March 30, 2020. This was a retrospective study. Written informed consent was sought from the patients’ parents or legal guardians. Clinical Trials Registry number: ChiCTR2000034910.

Registration URL: http://www.chictr.org.cn/showproj.aspx?proj=56726.

## Background

Unilateral coronal synostosis (UCS) is the premature fusion of one coronal suture, is also known as anterior or frontal synostotic plagiocephaly, and is rare, with an incidence of 1/10,000 live births [1]; in addition, UCS is the third most common type of simple craniosynostosis, preceded by involvement of the sagittal and metopic sutures [2]. UCS alters orbital development and predisposes patients to ocular disorders such as strabismus, astigmatism, and amblyopia [3, 4]. It is highly recommended that all patients who suffer from craniosynostosis be regularly examined by an ophthalmologist at the time of diagnosis and before and after craniofacial surgery [5]. The purpose of this investigation was to describe the strabismus and evaluate the outcome of surgical treatment. Another purpose of the study was to characterize the refractive error and other ophthalmic diseases in UCS patients.

## Methods

This was a retrospective cohort study based on ophthalmic data of patients with UCS recorded at the Department of Ophthalmology, Shanghai Children’s Hospital. All patients had radiographically confirmed UCS. Any patients with additional synostosis or other craniofacial abnormalities were excluded.

UCS was diagnosed on the basis of the clinical ophthalmic manifestation: recession and elevation of the ipsilateral superior orbital margin, elevation of the ipsilateral eyebrow and eyelid with contralateral ptosis [2, 6].

We reviewed 30 patients aged from 3 months to 6 years (median 1.8 years) who underwent UCS surgery at the Cerebral Department between 2017 and 2019. All patients had radiographically confirmed UCS. Inclusion criteria required a complete medical history, surgical treatment by fronto-orbital advancement (FOA) [4] and regular post-operative ophthalmological examinations. Patients were excluded for syndromic diagnosis, multi-suture coronal synostosis involvement, previous outside interventions, and incomplete ophthalmological follow-up.

Cycloplegic refractions were performed after coronal synostosis surgery. The coordinating technician administered drugs in the following manner: each eye received 5 drops of tropicamide phenylephrine 0.5%, and each drop was separated by 5 min. Cycloplegic refractions were obtained 30 min after instillation of tropicamide.

Demographics, cycloplegic refraction, ocular motility, and records of craniofacial and ophthalmic operations were referred during each clinic visit. Amblyopia was defined as a fixation preference. “Ipsilateral” and “contralateral” referred to the side of coronal synostosis.

All refractions were converted to the minus cylinder prescription. The axis of the cylindrical component was categorized as ‘with the rule’ if the minus cylinder axis 180° ± 15°, as ‘against the rule’ if it was 90° ± 15°, or as oblique if it was 45° ± 15°. Aniso-astigmatism was calculated for each patient as the absolute value of the minus cylinder of the ipsilateral eye minus the contralateral eye, despite the axis. Spherical anisometropia was considered the difference between binocular spherical equivalent.

**Fig. 1:**
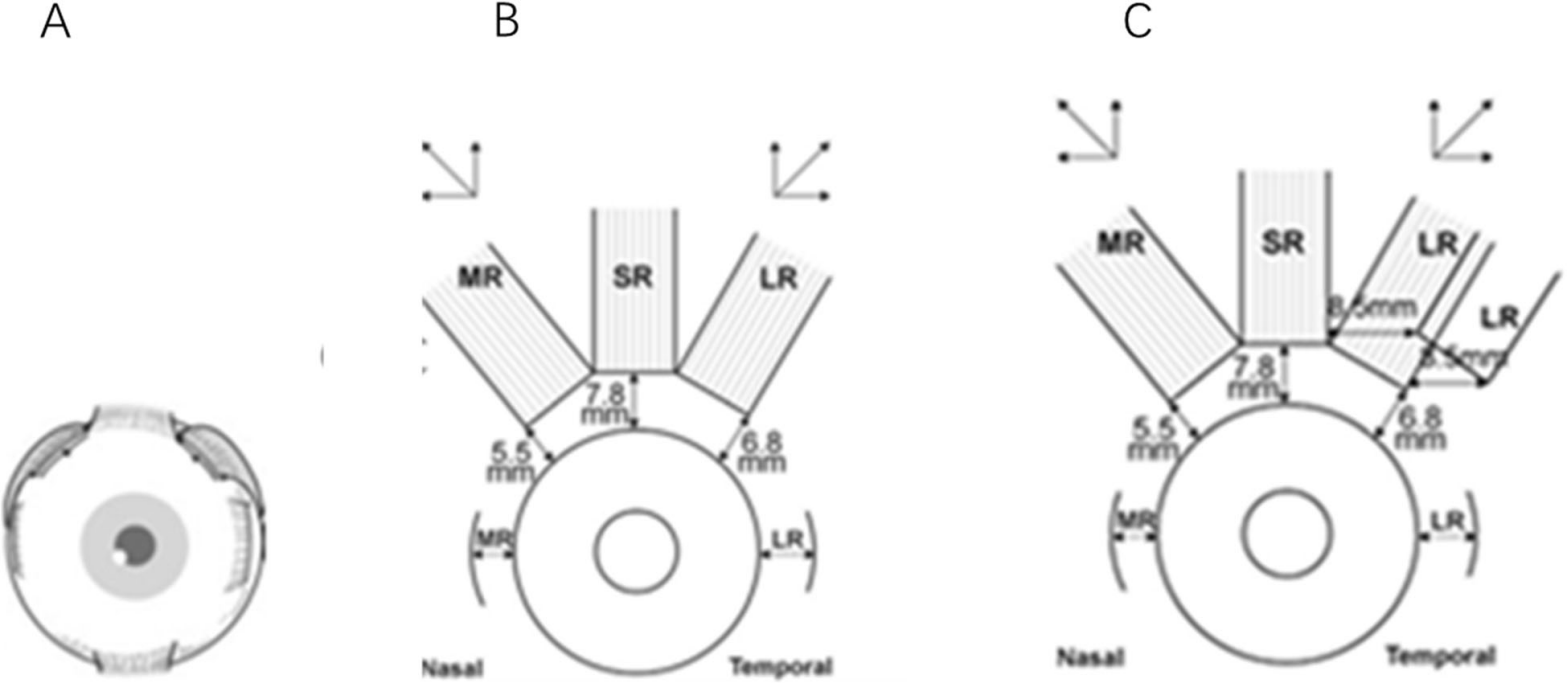
Standard Knapp procedure. **a**&**b**: MR: Medial rectus muscle; SR: Superior rectus muscle; LR: Lateral rectus muscle. **c**: Standard Knapp procedure with lateral rectus recession

Sixteen UCS patients with strabismus were recruited from Shanghai Children’s Hospital. One of 16 strabismus patients had not been offered the procedure because he had not yet reached the appropriate age for surgery. Fifteen strabismus patients aged from 1 year and 7 months to 6 years (median 2.7 years), including 10 males and 5 females, underwent procedures from January 2018 to December 2019. Due to an age not reaching the indication for operation, another strabismus patient did not receive the procedure. Six patients were diagnosed with monocular elevation deficiency (MED), their forced duction test (FDT) results were negative, and they underwent the standard Knapp procedure (Fig. 1a and b), with or without a horizontal deviation procedure (Fig. 1c). One patient was diagnosed with severe esotropia and underwent bilateral medial rectus recession. Due to the presence of congenital esotropia, vertical deviation was difficult to examine before the procedure. The possibility of concealed vertical deviation could not be ruled out. Only one of the patients seemed to have mainly horizontal deviation, and the remaining patients primarily had vertical deviation. Seven of 15 patients had congenital superior oblique palsy in at least one eye. Five of 7 patients were diagnosed with V pattern strabismus. Six patients were diagnosed with MED. One patient with trichiasis underwent a trichiasis procedure. One patient with contralateral congenital lacrimal obstruction underwent a probing operation.

Neutralizing prisms were held in front of each paralysed eye to measure the primary deviation. The level of motility anomaly was recorded as + 1 or more of muscle overaction and − 1 or more of under-action. FDT was performed after general anaesthesia using a non-depolarizing muscle flaccidity in all MED patients.

We analysed the ocular alignment and elevation improvement. The criterion of success was defined as a residual vertical squint ≤10 PD and ≥ 1 over-action or under-action improvement after the surgery. The follow-up ranged from 1 to 12 months (median 3.5 months).

## Statistical analysis

Statistical analysis was performed using IBM SPSS Statistics version 21. The variables, where appropriate, are reported as the mean and standard deviation. The two parameters of pre-operation and post-operation vertical deviation in MED patients were normally distributed, performed by applying the parametric paired sample T test. A non-parametric Wilcoxon signed-rank test was used to pairwise compare between the pre-operative and post-operative values. A *P* value of < 0.05 was considered significant.

## Results

**Fig. 2:**
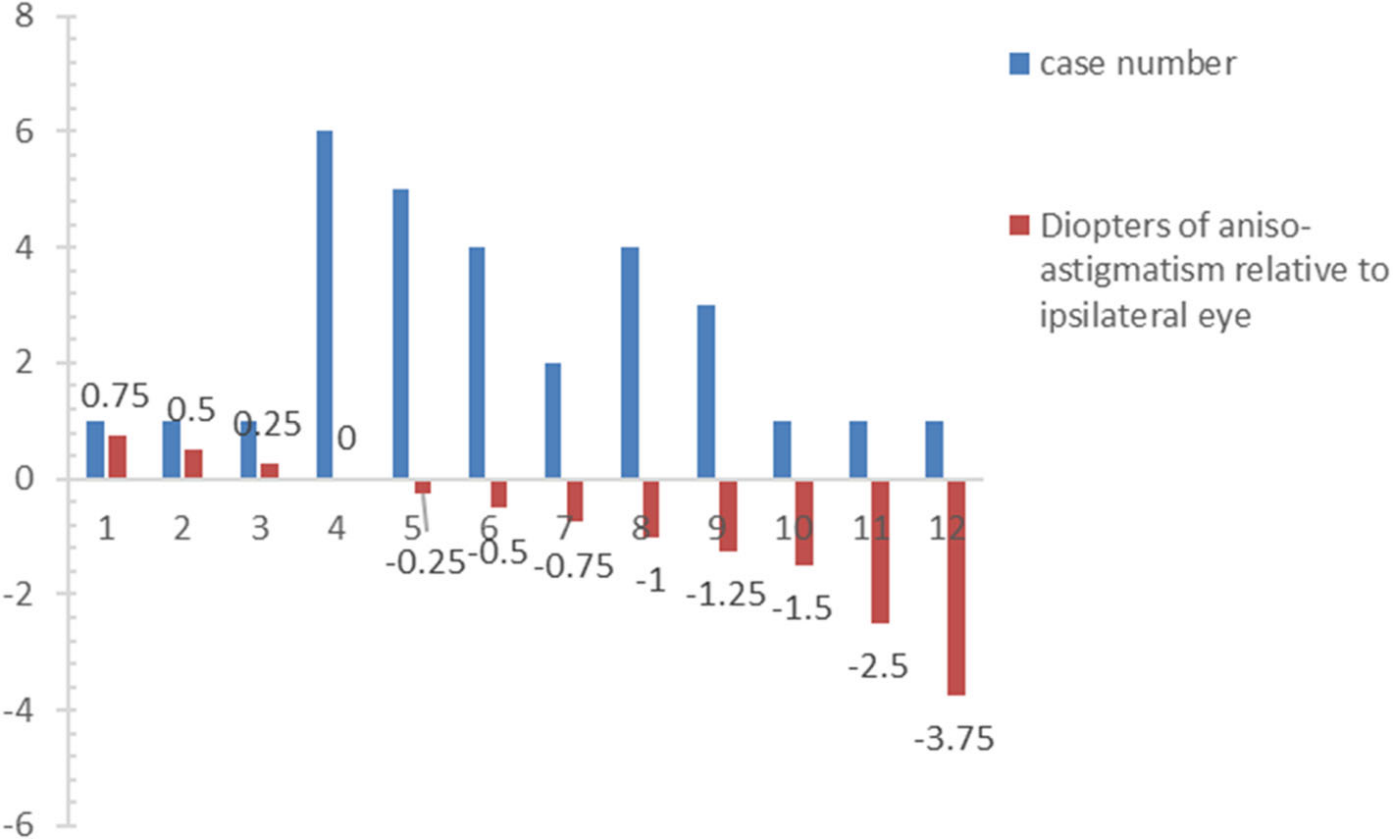
Histogram showing aniso-astigmatism at the last recorded refraction for each patient. Negative numbers indicate more astigmatism on the contralateral side to the synostosis

Refractive errors of thirty UCS patients were included in the study. A total of 43.3% (13/30) were female, and 70% (21/30) had a right-sided UCS. The median age at the last recorded refraction was 1.8 years (range, 3.3 months to 6 years). Sixty percent (18/30) of patients had 1.00 D or more astigmatism in not less than one eye at their last recorded refraction. Ten of the 18 (55.6%) had aniso-astigmatism of 1.00 D or more. Of these 18 patients, 8 (44.4%) had higher (1.00 D or more) astigmatism in the contralateral eye. Twenty of 30 (66.7%) patients had higher (0.25 D or more) astigmatism in the contralateral eye, while the other 3 patients had higher astigmatism in the ipsilateral eye, with aniso-astigmatism less than 1.00 D. The aniso-astigmatism of 30 patients is demonstrated in Fig. 2.

Table 1 shows the refraction error and ophthalmic problems in all eyes before strabismus surgery. Table 2 shows the axes of astigmatism. In both ipsilateral eyes and contralateral eyes, astigmatism was found most frequently ‘with the rule’; nevertheless, some likewise represented oblique axes. Spherical anisometropia may cause amblyopia, which was also calculated for each patient’s last refraction record. Five patients had not less than 1.00D spherical anisometropia.

**Table 1:** Refractive dioptres and ophthalmic problems before strabismus surgery

Case No.	Sex	Age	Ophthalmic Diagnosis	Unilateral Coronal Synostosis	OD Refraction	OS Refraction
1	female	2 y 3 mos	LMED	right	+ 0.75–1.25*180	+ 3.00–2.50*180
2	male	1 y 7 mos	LMED	right	+ 2.50–1.25*180	+ 3.25–1.75*180
3	male	2 y 7 mos	LMED	right	+ 3.50–2.00*155	+ 1.75–3.00*175
4	male	1 y 10 mos	LMED	right	−1.00-0.50*165	−2.25 − 0.50*135
5	female	2 y	Cong ET	right	+ 4.25–1.25*140	+ 4.75–0.50*165
6	male	5 y 2 mos	LMED& IXT	right	0	0
7	male	4 y 4 mos	LSOP&R trichiasis	left	+ 2.25–1.50*20	+ 1.00–0.50*180
8	male	2 y	CXT	left	-0.50-0.25*145	0
9	male	7 mos	IXT	right	+ 2.00–0.33*59	+ 2.50–1.33*179
10	male	9 mos		right	+ 0.75–1.00*160	+ 1.00–1.50*30
11	male	3 mos		right	+ 0.75–1.25*15	+ 1.00–2.50*5
12	female	6 mos	LMED	right	+ 0.50–0.75*180	+ 1.00–1.00*180
13	female	4 y 6 mos		right	+ 2.25–0.25*180	+ 2.50–1.50*180
14	male	2 y 1 mo		left	+ 3.50–4.50*11	+ 0.50–0.75*5
15	female	1 y 2 mos		right	+ 0.50–1.75*180	+ 0.50–1.75*180
16	male	5 mos 4 d		right	+ 0.50–0.50*180	−1.25*180
17	male	1 y 7 mos		right	+ 0.25–0.50*180	+ 0.75–3.00*180
18	female	8 mos	LMED	right	+ 0.50–0.75*180	+ 0.50–0.50*180
19	female	10 mos	LCLDO	right	+ 0.75–0.50*180	+ 0.75
20	female	20 mos		right	0	+ 0.25
21	female	9 mos		right	+ 0.25–0.25*132	+ 0.50–0.50*74
22	male	1 y 3 mos		right	+ 0.25	+ 0.25–1.00*180
23	female	4 y 7 mos	VXT	left	+ 2.00–1.00*5	+ 1.25–0.50*2
24	male	2 y 6 mos	VXT	left	−0.50 − 0.25*145	0
25	female	5 y	VXT	right	-0.25-0.50*110	+ 1.50–0.75*5
26	female	3 y 2 mos	LSOP&IXT	left	+ 1.00–0.75*180	+ 1.25–0.75*180
27	female	3 y 1 mo	VXT	left	+ 3.25–1.75*43	+ 2.50–0.25*153
28	male	2 y 8 mos	RMED	left	+ 1.00–1.25*180	+ 1.00–0.50*180
29	male	6 y	VET	right	+ 1.50–0.75*90	+ 0.75–0.75*180
30	male	5 y	VXT	left	+ 2.00–0.75*175	+ 1.25–0.25*150

*LMED* left MED, *Cong ET* congenital esotropia, *IXT* intermittent exotropia, *CXT* constant exotropia, *LSOP* left superior oblique palsy, *VXT* V pattern exotropia, *VET* V pattern esotropia, *LCLDO* left congenital lacrimal duct obstruction

**Table 2:** Axes of astigmatism at the last recorded refraction for eyes ipsilateral and contralateral to the suture

	Eye numbers	
	Ipsilateral	Contralateral
With the rule	17	22
Against the rule	2	3
Oblique	6	2
No astigmatism	5	3

Because UCS children have a high incidence of strabismus and refractive error, we called them back to the ophthalmology clinic for a regular eye examination for refraction and ocular alignment half a year after UCS surgery. We have listed the last refraction values before the strabismus surgery in Table 1.

Among the 30 UCS patients, 15 strabismus patients underwent surgery, including 6 with contralateral MED, 1 with exotropia, 2 with unilateral superior oblique palsy, 5 with V pattern deviation (bilateral superior oblique palsy) and 1 with esotropia. Observations of the horizontal or vertical squint and elevation deficiency or overaction were compared pre- and post-operatively, as shown in Table 3. We also surveyed age, ocular alignment, coronal synostosis, operation and follow-up periods (Table 3).

**Table 3:** Pre- and post-operative evaluations

Case No.	Age	Eye position	Deviation (PD)				Procedure	Elevation deficiency			F/U (mo.)
			Pre-op.	Post-op.	Correction	UCS		Pre-op.	Post-op.	Correction	
1	2 y 3 mos	OS hypo	-30R/L25	0	25	R	Left Knapp	2-	0	2	12
2	4 y 4 mos	OS hypo	L/R20	0	20	L	LIOA+R lower eyelid trichiasis	LIOO2+	0	2	5
3	5 y 2 mos	OS hypo	-40R/L20	0	20	R	Left Knapp RLRR+LLRR	2-	0	2	4
4	2 y 7 mos	OS hypo	R/L30	0	30	R	Left Knapp	2-	0	2	2
5	1 y 7 mos	OS hypo	R/L30	0	30	R	Left Knapp	2-	0	2	2
6	1y 10 mos	OS hypo	R/L30	5	25	R	Left Knapp	2-	1-	1	7
7	2 y	esotropia	80	0	80	R	RMRR+LMRR	0	0	0	3
8	2 y	exotropia	-50	0	50	L	RLRR+LLRR	0	0	0	6
9	2 y 6 mos	V pattern exotropia	-80	0	80	L	RLRR+LLRR+IOA	LIOO4+RIOO+	0	R4 L1	1
10	5 y	V pattern exotropia	-15	0	15	R	RIOA+LIOA	RIOO3+LIOO2+	RIOO1+	R2L2	3
11	3 y 2 mos	OS hyper exotropia	-35	-5	30	L	RLRR+LLRR+LIOA	LIOO2+	0	2	7
12	4 y	V pattern exotropia	-40	0	40	L	RLRR+RIOA+LLRR+LIOA	RIOO1+LIOO1+	0	1	7
13	2 y 8 mo	OS hyper	L/R20	R/L10	20	L	Right Knapp	2-	1+	3	7
14	6 y	V pattern esotropia	15	0	15	R	LMRR+RIOA+LIOA	RIOO2+LIOO2+	0	2	1
15	5 y	V pattern exotropia	-60	0	60	L	LLRR+RIOA+LIOA	RIOO2+ LIOO4+	LIOO1+	R2L3	3

*PD* Prism dioptre, *Hypo* Hypotropia, *HYPER* Hypertropia, *RLRR* Right lateral rectus recession, *LLRR* Left lateral rectus, *RMRR* Right medial rectus recession, *LMRR* Left medial rectus recession, *LIOO* Left inferior oblique overaction, *IOA* inferior oblique anteriorization. Bell’s sign was positive in all patients. FDT was negative in all six MED patients

We checked the vertical squint pre-operatively, at the time of surgery and post-operatively in MED patients. All three parameters were normally distributed, indicating that parametric tests could be used.

In six MED patients, the vertical squint was diminished in the primary position from 25.83 Δ ± 4.92 Δ (range, 20 Δ-30 Δ) to 0.83 Δ ± 4.92 Δ after surgery (range, 0 Δ-10 Δ), for an improvement of 26.67 Δ ± 4.08 Δ (t = 16 *P* < 0.05).

The elevation deficiency improved from − 2 to 0 (0.50) for an improvement of 2 (0.50) units post-operatively (*Z* = 2.264 *P* < 0.05). Of these 15 strabismus patients, 5 V patterns and 2 superior oblique palsy patients showed vertical deviation. Elevation overaction is probably overaction of the inferior oblique. The elevation overaction improved from + 2 (1.5) to 0 after surgery for an improvement of 2 (0.75) units. (*Z* = -3.133 *P* = 0.002).

**Fig. 3:**
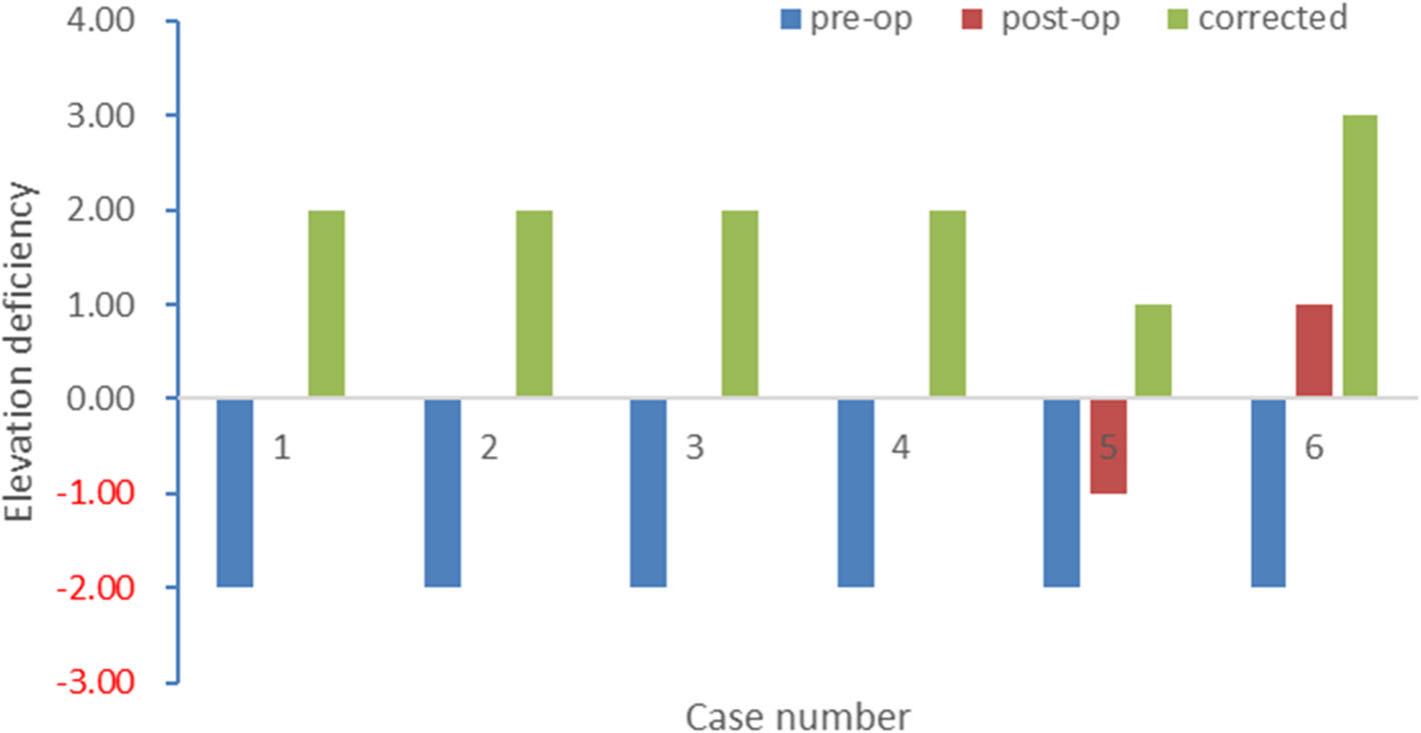
The corrected elevation deficiency changes pre- and post-operatively in six double elevator palsy cases

The details of elevation deficiency changes pre- and post-operatively are shown in each case (Fig. 3).

Six patients were diagnosed with MED, in whom the FDT was negative and Bell’s sign was positive. In the six patients, the sound eye was dominant. The other 2 patients had ipsilateral inferior oblique palsy, one of them with contralateral trichiasis, 4 with V pattern exotropia, 1 with V pattern esotropia, one with esotropia, and one with exotropia. At the follow-up visit after surgery, all 15 strabismus operations (100%) succeeded.

**Fig. 4:**
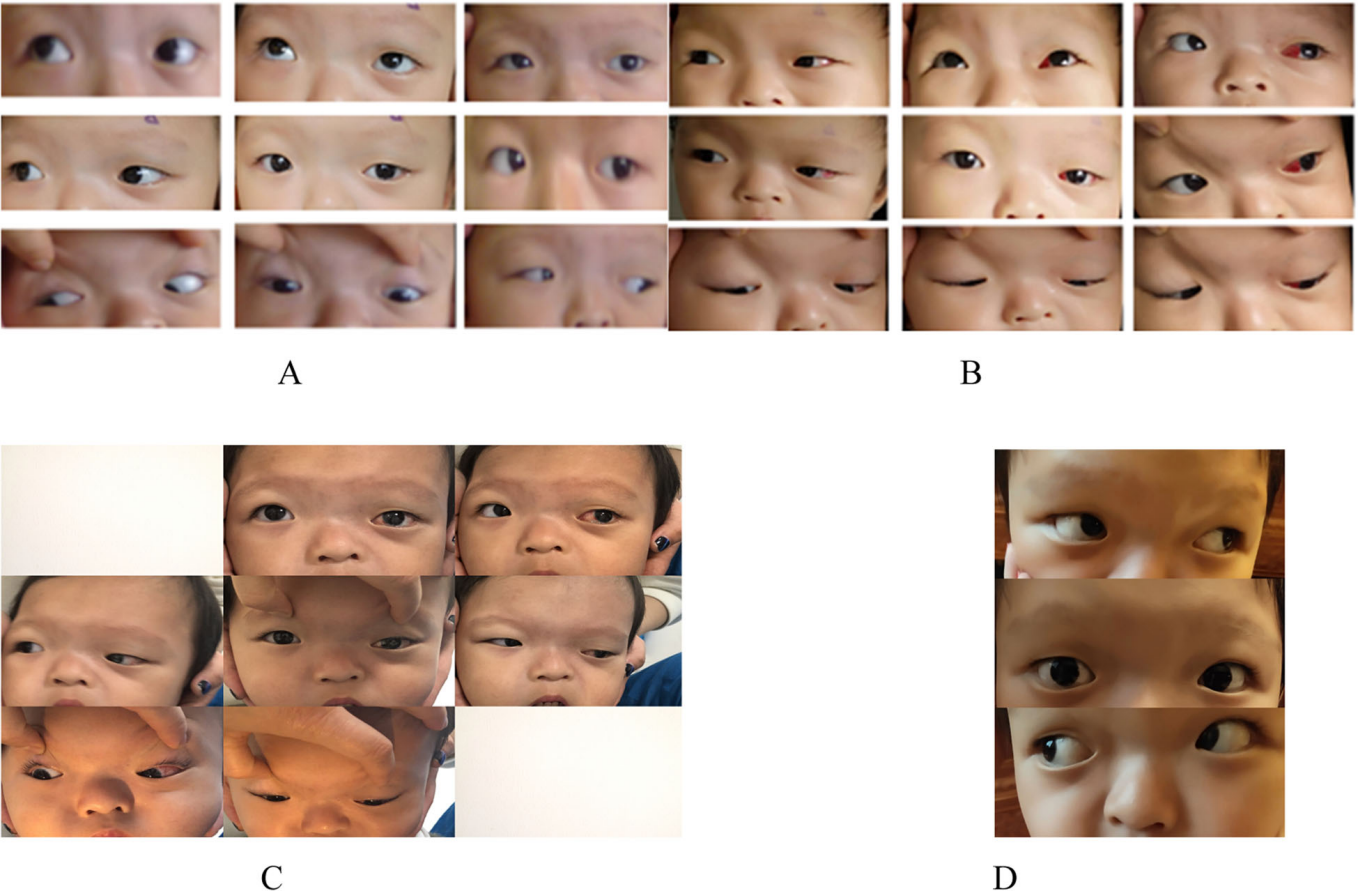
**a**: Nine gaze positions of a right UCS patient showing deficient elevation of the left eye in both adduction and abduction; **b**: 1 day after standard Knapp surgery, the eye elevation improved significantly post-operatively. **c**: 1 month after standard Knapp surgery. **d**: 1 year after standard Knapp surgery

A right UCS patient aged 1 years and 7 months showed deficient elevation of the left eye in both adduction and abduction pre-operatively (Fig. 4a). The elevation deficiency had improved in one day after the Knapp procedure (Fig. 4b). At the one-month and one-year follow-ups after the operation, the elevation deficiency had improved significantly compared with that in the pre-operative period. The follow-up period lasted 1 year (Fig. 4c & d).

**Fig. 5:**
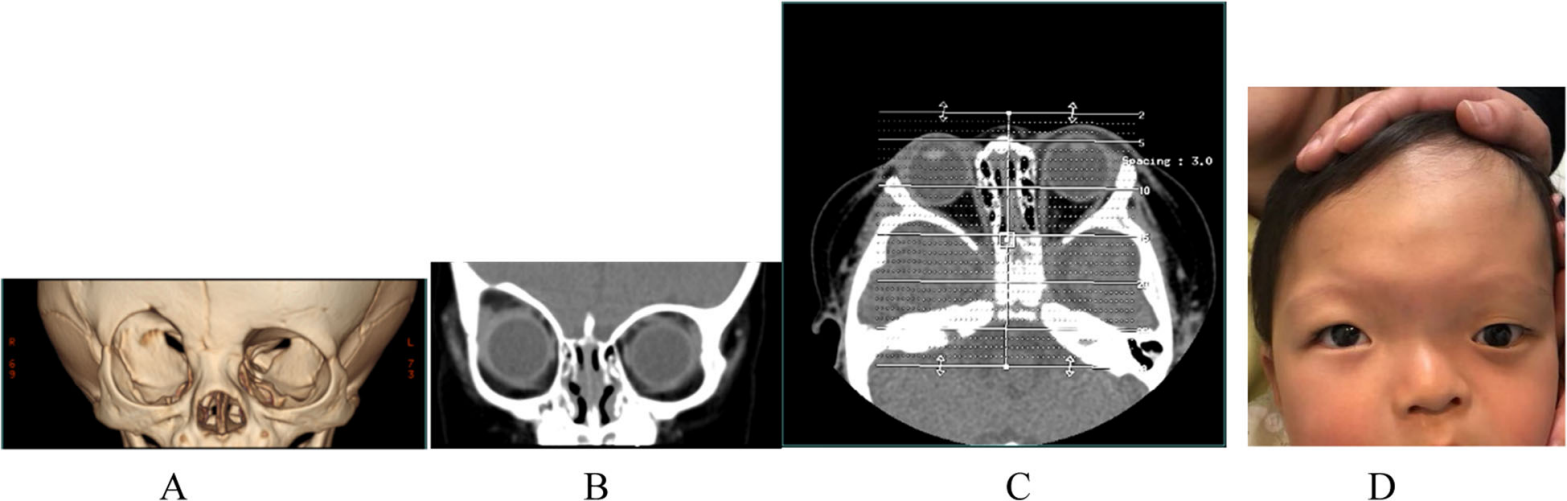
**a** The three-dimensional reconstruction of the computed tomography (CT) scan of the child’s skull with right UCS; **b**: Coronal view; **c**: Axial view; **d**: Right UCS with LMED

Figure 5a &b &c shows malformation of the UCS obit, recession and elevation of the ipsilateral superior orbital rim. One right UCS skull before the craniotomy is shown in Fig. 5a & b. The craniofacial malformation was caused by premature closure of the right coronal suture.

**Fig. 6:**
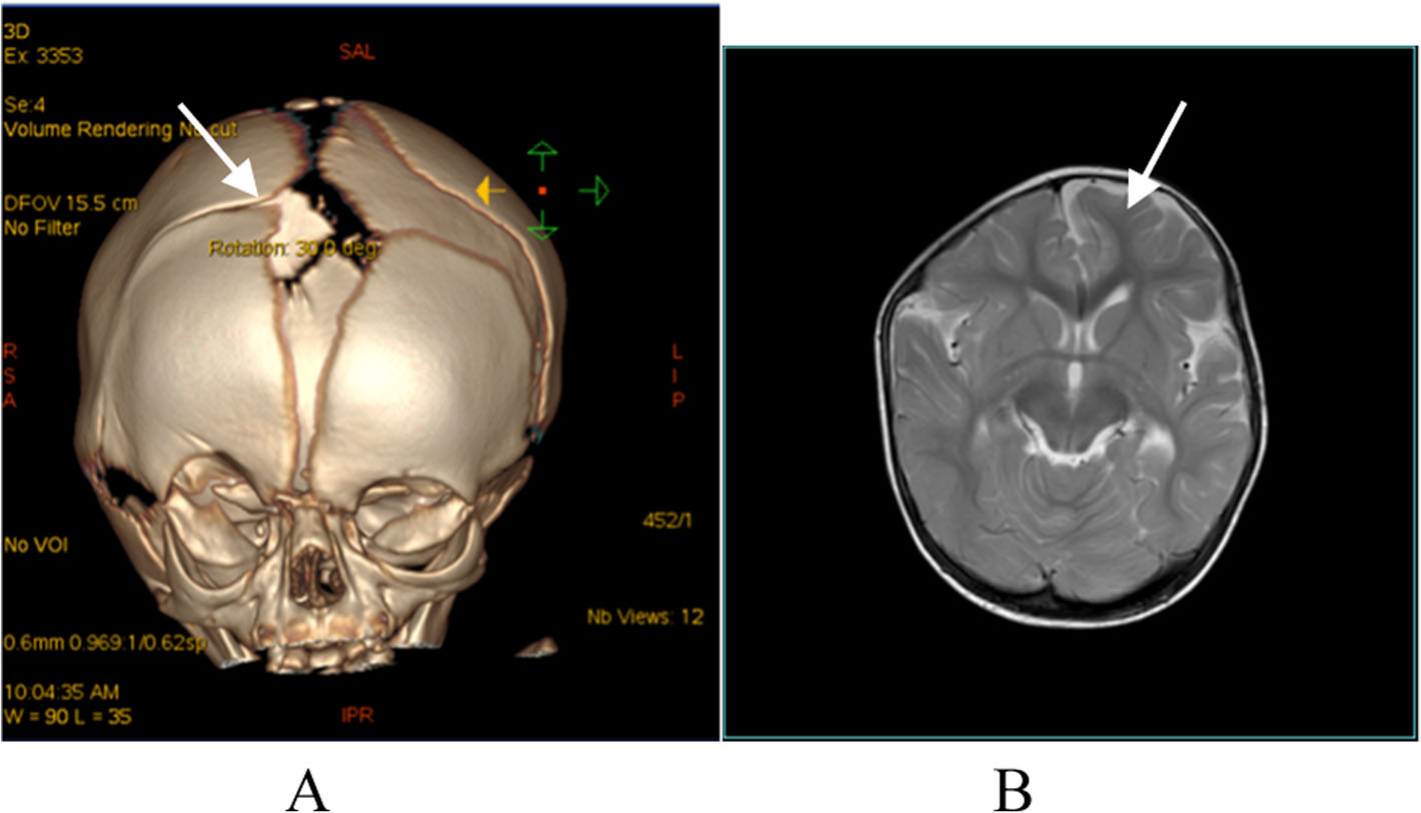
**a** Three-dimensional reconstruction of a computed tomography (CT) scan of a child’s skull with right UCS after craniofacial surgery. The white arrowhead refers to the right coronal synostosis. 7-**b**: Cerebral magnetic resonance imaging of another child with right UCS

The skull malformation of the child with right UCS is shown in Fig. 6a. After craniofacial surgery, the frontal bone shifted forward. Figure 6b Cerebral magnetic resonance imaging of the right UCS shows an asymmetric brain, with the ipsilateral hemisphere significantly smaller than the contralateral hemisphere.

**Fig. 7:**
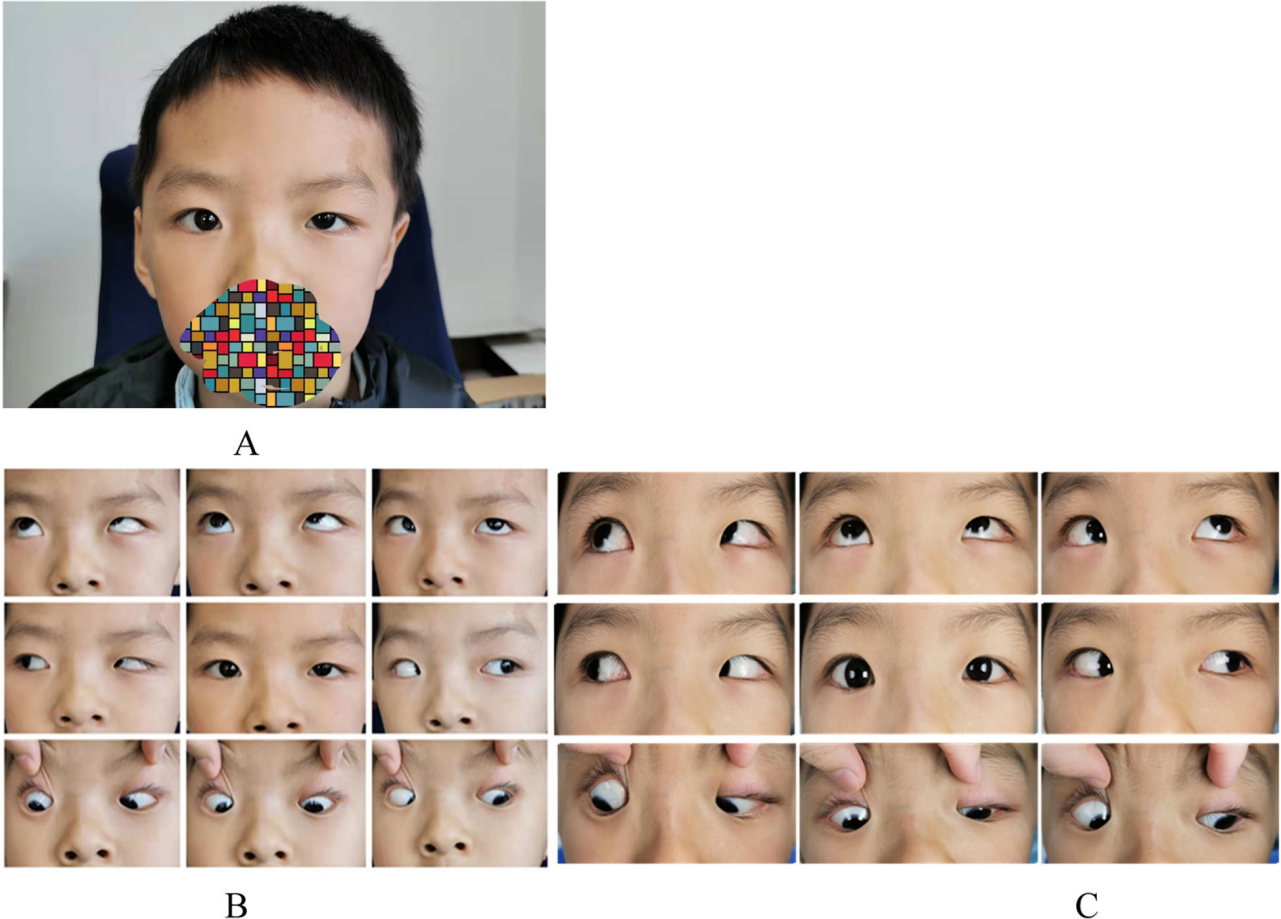
**a**: A six-year-old right UCS patient with esotropia V pattern strabismus. **b**: Overaction elevation of both eyes is shown in adduction pre-operatively; **c**: 1 month after surgery, the eye overaction elevation was recovered significantly post-operatively

Many doctors cannot recognize UCS. The child who had right UCS with V pattern esotropia underwent craniofacial reconstruction until half a year ago, when he was already more than 5 years old. His skull was obviously asymmetrical (Fig. 7a). He had undergone bilateral medial rectus recession and inferior oblique anteriorization procedures when he was 6 years old. We successfully corrected the horizontal and vertical deviations simultaneously. Overaction elevation of both eyes was shown pre-operatively in adduction (Fig. 7b). By the 1-month follow-up after strabismus surgery, the elevation overaction had recovered (Fig. 7c). The follow-up periods lasted for 1 month. No obvious complications occurred after strabismus surgery.

## Discussion

Premature closure of one of the cranial sutures results in a restricted growth pattern across the fused suture, while a compensatory or accelerated growth pattern typically occurs parallel to the affected synostosis [7]. Fibroblast growth factor receptor (FGFR) mutations are most frequently cited in association with craniosynostosis, especially syndromic types. A single genetic anomaly has still not been identified as a cause for craniosynostosis [8]. UCS children usually present with the characteristic signs involving the ipsilateral side: (1) Flattening of the forehead and a shallow orbit, resulting in an ipsilateral increased vertical diameter; and (2) tilting of the head as a compensatory mechanism resulting from both the extraocular muscle imbalance [9]. UCS not only affects one coronal suture but also affects orbital skeletal development. There are many disorders, including eyelid anomalies, ptosis and trichiasis, strabismus, proptosis, and refractive error [10]. Many patients experience severe forms of the disease, causing a significant impact on their quality of life. We treated all ophthalmic complications after cranioplastic surgery [11], even after fronto-orbital advancement (FOA) [12], although many reports summarize that FOA does not seem to shorten rates of strabismus [4, 10].

There is a great difference between healthy children and children with UCS in the prevalence of strabismus. Chen et al [13] reported that the prevalence of strabismus in a population-based sample of preschool children aged 36–72 months in eastern China was 5.65% (95% CI 5.05 to 6.25%). Intermittent exotropia was the most common type of strabismus among all children (57.81, 95% CI 49.48 to 66.14%); however, pure vertical strabismus was unusual (3.13%). Matsuo [14] et al reported that the prevalence of strabismus was 1.28% (95% CI 1.24 to 1.36%) in Japanese elementary school children, and the prevalence rates of any types of exotropia and esotropia were 0.69 and 0.28%, respectively.

In this investigation, there were 9 cases of simple strabismus and 6 cases of complex strabismus with other ocular complications. Strabismus patients included 6 with contralateral MED, 5 with V pattern strabismus, 1 with exotropia, 1 with esotropia and 2 with superior oblique palsy. We found that vertical deviation was more common than exotropia, possibly due to an abnormal orbital bone.

One patient with trichiasis underwent a trichiasis procedure. One patient with contralateral congenital lacrimal duct obstruction underwent a probing operation.

Due to a young age, 1 child with strabismus had not been offered strabismus surgery. One 5-month-old patient with right UCS showed 40 Δ esotropia deviation and hypermetropia of 5.00 dioptres in both eyes. Fully correcting refractive error and occlusion is the initial treatment. We will await a good opportunity for the procedure.

Both UCS and double elevator palsy are rare diseases. Contralateral MED was also the main type of strabismus in UCS. Superior oblique muscle palsy was still the most common as previously reported (74–100%) [10], and the ipsilateral eye was more frequently involved (≥32 to 100%) [10]. Nischal speculated that changes in the orbital shape and axes changed the oblique muscle insertions on the synostotic side, mimicking superior oblique paresis and thus creating hypertropia (manifesting as a deviation of one eye in an upward direction). Additionally, the trochlea is displaced for the orbital rotation and relative recession of the frontal process in UCS, causing a change in the angle through which the superior oblique turns, creating a mechanical disadvantage for its action [15]. Some studies have reported a predominance of esotropia (60–100%) [4, 16–18] and exotropia (36%) [19] in their patient populations. Our paper is the first to discuss MED in UCS; strabismus surgery must be performed after craniofacial reconstruction because reducing intracranial pressure is the most important treatment for saving lives. The hypotropia in the primary position is contralateral to the affected synostosis and increases in elevated gaze (adduction and abduction), with apparent under-action of the contralateral inferior oblique and superior rectus muscles. Therefore, hypotropia is treated in a surgically similar manner to routine MED. MED is defined as the inability to elevate one eye equally in abduction, adduction, caused by paralysis of the superior rectus and inferior oblique [20]. Knapp [21] created the traditional Knapp procedure (Fig. 1a). Because of the rotation of the eye caused by the traditional procedure, we adopted the standard Knapp procedure: the medial rectus and lateral rectus muscles were transposed superiorly to the insertion of the superior rectus muscle (Fig. 1a & b). We reported the management of double elevator palsy patients with standard Knapp procedures or augmented Knapp procedures in 2018 [22].

Many reports support ipsilateral retrusion of the forehead and elevation of the superior orbital margin; with widening of the palpebral fissure; the contralateral side develops compensatory bossing of the forehead and narrowing of the palpebral fissure [2, 6]. Joel [23] provided evidence that both orbits in UCS patients are dysmorphic. The ipsilateral orbit is tall and narrow in morphology and smaller in volume, whereas the contralateral side is vertically short and wide in morphology and larger in volume. This contrasts with unaffected individuals who have a good deal of orbital symmetry in both volume and morphology. As orbital asymmetry may form the basis for many of the ocular abnormalities associated with UCS, bilateral orbit reconstruction should be considered. We propose two hypotheses about MED. First, the FGFR mutation may lead to deformation of the extraocular muscle (EOM) and orbital shape. Second, we hypothesize that the skull deformation and orbital deformation results in changes in muscle active road and strength. We have chosen to obtain evidence of anatomical support in the near future.

According to the latest technology, even 3D images cannot provide obvious evidence to support our hypothesis. In all UCS cases, we found no deformation of EOM, unlike Crouzon or Pfeiffer syndrome. EOM insertion dislocation, lack of muscle, and weak musculature always exist in Crouzon or Pfeiffer syndrome. Although we found no abnormalities in the pulley and muscles of the contralateral eye, we speculated that contralateral dysmorphic orbit or unilateral supranuclear lesions in the pretectal area near or inside the third cranial nerve nucleus [24] may cause MED.

V pattern exotropic strabismus is common in patients [25] with craniofacial dysostosis, with as many as two-thirds of patients manifesting the condition, which is similarly common in UCS patients. This is our first report on the management of V pattern horizontal strabismus in UCS.

Many papers have discussed the long-term visual outcomes after craniofacial surgery in all kinds of craniosynostosis [26–29], and few papers have shared experience with lid abnormalities in cases of craniosynostosis.

Because of apoptosis and the lesser amount of orbital fat pad, trichiasis in the lower lid always reduces the quality of life in UCS, which hurts the cornea leading to photophobia, red eye, and tilt head position. The Hotz method was adopted, and we removed a strip of skin and orbicularis oculi muscle and sutured the skin with the lower tarsus. Tarsus in children with craniosynostosis was thinner than normal. Therefore, scar tissue was the main strength of ectropion. Surgeons must be careful to suture the thin tarsus preventing perforation, and a 6–0 absorbable suture is our first choice.

Many papers have focused on the treatment of refractive errors and amblyopia. In our papers, we found a considerably high occurrence of astigmatism in the contralateral eye. Richard et al [30] speculated that this is caused by the inferior displacement of the superior orbital margin and roof, which possibly impacts the corneal curvature; the slightly increased globe volume may exacerbate this phenomenon.

## Conclusions

UCS is a complex disorder, and management requires coordinated effort from a multidisciplinary team. Contralateral MED was also the main type of strabismus in UCS. Superior oblique muscle palsy was still the most common, as previously reported. The vertical deviation was less than 30 PD in all MED patients, so we performed a standard Knapp procedure. We performed inferior oblique muscle anteriorization to correct superior oblique palsy and achieved success in all strabismus surgeries. Patients are at risk for developing a greater degree of astigmatism and anisometropia in the eye contralateral to the synostosis. Saving eyesight and recovering visual function are the goals of all interventions after all surgeries, correcting refractive errors and training amblyopia in the long-term period. Other ocular disorders should be treated in a timely manner.

**Additional file 1**

## Data Availability

Supporting data can be accessed by contact with the corresponding author (qiao joel@163.com). The datasets analysed are available from the corresponding author upon reasonable request. We could provide the video of the operation.

## Abbreviations

UCS: Unilateral coronal synostosis
MED: Monocular elevation deficiency
FDT: Forced duction test
FOA: Fronto-orbital advancement
PD: Prism dioptre
Hypo: Hypotropia
HYPER: Hypertropia
LRR: Lateral rectus recession
MRR: Medial rectus recession
LIOM: Left inferior oblique muscle
LIOO: Left inferior oblique overaction
IOA: Inferior oblique anteriorization
